# Haematopoietic stem cell transplantation in systemic sclerosis

**DOI:** 10.1136/rmdopen-2017-000533

**Published:** 2018-06-18

**Authors:** Ulrich A Walker, Lesley Ann Saketkoo, Oliver Distler

**Affiliations:** 1 Department of Rheumatology, University Hospital Basel, Basel, Switzerland; 2 Tulane University School of Medicine Lung Center, New Orleans Scleroderma and Sarcoidosis Patient Care and Research Center, University Medical Center – Comprehensive Pulmonary Hypertension Center, New Orleans, Louisiana, USA; 3 Department of Rheumatology, University Hospital Zurich, Zurich, Switzerland

**Keywords:** systemic sclerosis, treatment, pulmonary fibrosis

## Abstract

Three randomised controlled trials of haematopoietic stem cell transplantation (HSCT) in systemic sclerosis (SSc) demonstrated long-term survival benefits, induction of clinically meaningful, sustained improvement of forced vital capacity with improvements in skin thickening, vasculopathy and health-related quality of life, in contrast to a clinical decline in standard of care control groups. These benefits, however, must be weighed against the increased risk of transplant-related mortality. Further, with disease progression, severe extensive internal organ involvement and damage ensues, constituting an exclusion criterion for safety reasons, leaving a limited window whereby patients with SSc are eligible for HSCT. Although autologous HSCT offers the possibility of drug-free remission, relapse can occur, requiring re-initiation of disease modifying antirheumatic drugs. HSCT is also associated with secondary autoimmune diseases and gonadal failure. HSCT should be proposed for carefully selected patients with early rapidly progressive diffuse SSc whose clinical picture portends a poor prognosis for survival, but yet lacks advanced organ involvement.

**Key messages**

- Autologous haematopoietic stem cell transplantation (HSCT) is currently the only disease modifying strategy that demonstrated grade A evidence for improving long-term survival, prevention of organ worsening as well as improvement of skin and pulmonary function in systemic sclerosis (SSc).
- A limited window of opportunity exists for HSCT treatment in SSc as severe irreversible organ involvement precludes transplantation.
- Autologous HSCT should be considered for carefully selected patients with early rapidly progressive diffuse SSc and a poor prognosis for survival.
- Risks of HSCT include, but are not limited to, early treatment-related mortality, gonadal failure and secondary autoimmune diseases.

## Introduction

Systemic sclerosis (SSc) is a rare, clinically heterogeneous multisystem autoimmune disorder driven by inflammation, fibrosis and a microangiopathic vasculopathy. Internal organ involvement greatly impacts physical and psychological functioning, impairing one’s ability to work and participate in social activities.^1^ SSc disease progression is the leading cause of patient all-cause mortality, largely related to end-stage organ involvement.^1 2^ Pulmonary and cardiac complications are the leading drivers of SSc-specific mortality.^2 3^

Current SSc treatment recommendations have, until recently, focused on the management of individual organ manifestations.^4 5^ High grade evidence from randomised placebo-controlled clinical trials (RCTs) for potential disease modifying agents currently exists only for methotrexate and cyclophosphamide. The clinical benefit of these drugs is, however, limited by moderate or short-term efficacy.^6–9^ Great hope anticipates several ongoing prospective phase II/III RCTs of targeted therapies in SSc, with results expected in 2018/2019.^10^

Haematopoietic stem cell transplantation (HSCT) has been used in the treatment of autoimmune disorders refractory to conventional immunosuppression for over 2 decades.^11^ Since 2013, the yearly frequency of HSCT has steadily increased,^12^ with the European Society for Blood and Marrow Transplantation (EBMT) registering approximately 2300 HSCT procedures in 2016 for a variety of autoimmune diseases.^12^ Three RCTs have now demonstrated clinically meaningful improvement of organ involvement and survival in patients with SSc undergoing HSCT, further increasing awareness and interest in this complex procedure.^13–15^ These results have sparked the European League against Rheumatism (EULAR) to recommend that HSCT be considered for patients with rapidly progressive SSc who are at risk for organ failure.^4^ The aim of this manuscript is to examine the rationale, benefits and risks of HSCT in SSc, predicated on the high-level findings produced from RCTs.

### Rationale

HSCT aims to non-specifically immunoablate aberrant self-reactive T-cells and B-cells via high-dose immunosuppression, with subsequent reconstitution of a renewed and tolerant immune system by means of infusing a patient’s previously collected haematopoietic stem cells (‘transplantation’).^16^ Autologous HSCT (employing the patient’s own stem cells) is preferred over allogeneic HSCT (employing stem cells from a different person), due to autologous HSCT having a lower treatment-related mortality and lack of graft-vs-host disease, although sustained remissions of SSc have been observed with both procedures.^17 18^

There are four main stages of HSCT. *Mobilisation*: Stem cells are stimulated and mobilised from the patients’ bone marrow using substances like granulocyte colony-stimulating factor (G-CSF) and cyclophosphamide which causes the release of proteases with cleavage of adhesion molecules, facilitating the release of haematopoietic stem cells into the peripheral blood; however, G-CSF alone has been shown to be efficacious in mobilisation. *Harvesting*: subsequently these stem cells are collected from peripheral circulation and stored in liquid nitrogen. At this point, either simple apheresis or *stem cell manipulation* with selection for CD34+ can occur (or if the patient’s umbilical cord blood was banked it can be used to supplement quantity). Though selection for and reinfusion of high concentration CD34+ cells may result in extended periods of severe immunodeficiency, such manipulation may prevent reinfusion of autoreactive cells. *Conditioning*: A few weeks following stem cell harvest, the majority of resident autoreactive T-lymphocyte and B-lymphocyte subsets are eliminated using high doses of cyclophosphamide, antithymocyte globulin (ATG) with/without total body irradiation (TBI). Conditioning regimens without TBI are predominantly lymphoablative, profoundly depleting lymphocytes, while preserving cyclophosphamide-resistant myelogenous stem cells, whereas regimens employing TBI are also myeloablative. *Transplantation*: shortly after conditioning, stem cells are thawed and reinfused. Endogenous haematopoiesis could reconstitute even without transplantation; however, stem cell grafting shortens the period of pancytopenia, allowing adaptive immunity to be rebuilt through clonal expansion of the remaining immunocompetent cells, formation of new non-autoreactive cells (thymopoiesis) and graft-derived regulatory T-cells.^16^ Complete elimination of autoreactive T-cells is impossible, but through clonal expansion they become outnumbered by the newer tolerant clones, with relatively few patients with autoimmune diseases relapsing despite persistent post-transplant autoreactive clones.^19^

### Benefits of autologous HSCT

Multiple cohorts^20–24^ and a case control study^25^ suggested beneficial effects of autologous HSCT in patients with rapidly progressive SSc. The majority of patients treated with HSCT had the early rapidly progressive diffuse cutaneous form of SSc, without yet having progressed to severe internal organ involvement.

In these SSc cohorts, HSCT predominantly induced a medically significant improvement of the modified Rodnan skin score (mRSS),^17 20 21 26 27^ forced vital capacity (FVC),^26^ extent of fibrosis on chest CT^28^ as well as both the physical and mental components health-related quality of life (HRQoL).^26 27^ Furthermore, HSCT rapidly improved skin fibrosis beyond that of the control group^20^ and unlike conventional treatment with cyclophosphamide^29^ reduced capillary loss, both of which are hallmark characteristics in the pathogenesis of SSc.^29–31^

Three months after the procedure, the mRSS had regressed from a mean of 27 to 5 in one cohort^17^ and from 24.1 before transplantation to 16.5 by 6 months and 12.9 by 12 months in another cohort.^20^ As these data were uncontrolled, the improvement of mRSS needs to be interpreted with caution.

SSc relapses after successful HSCT do occur. In an analysis of 57 patients with SSc who had undergone autologous HSCT, partial or complete responses were seen in 92% of patients, but 35% of patients relapsed within 10 months.^32^ The EBMT cohort calculated a 63% 3-year progression-free survival of patients with SSc after transplantation, and the overall survival after 3 years was 80%.^22^ Multivariate analysis indicated that progression-free survival rates were higher in younger than in older patients.^22^ In US cohorts, the 5-year progression-free survival was reported between 64% and 70% and the overall survival between 64% and 78%.^13 20^

Uncontrolled clinical studies, including cohort studies, are difficult to interpret in SSc because the natural disease course evolves with regression of skin fibrosis and overall disease activity over time. Thus, RCTs are of key importance to substantiate efficacy of a treatment regimen in SSc. Three RCTs of autologous HSCT have been performed. All three trials included patients with early forms of diffuse SSc and high risk of death from or developing internal organ involvement. Patients with severe internal organ involvement were excluded (table 1).

**Table 1:** Comparison of patient selection, treatment modality and outcomes among three randomised trials investigating HSCT in SSc.

	ASSIST^13^	ASTIS^14^	SCOT^15^
Patient number	19	156	75
Inclusion criteria	<60 years of age	18–65 years of age	18–69 years of age
	Diffuse SSc	Diffuse SSc	Diffuse SSc
	mRSS≥15	mRSS≥15	mRSS≥16
	Disease duration ≤4 years	Disease duration ≤4 years	Disease duration ≤4 years
	Internal organ involvement	Internal organ involvement	Internal organ involvement
Exclusion criteria	Mean PAP>25 mm Hg or PAPsys>40 mm Hg	Mean PAP>50 mm Hg	Mean PAP>30 mm Hg
	LVEF<40%	LVEF<45%	LVEF<50%
	–	–	FVC<45% predicted DLCO<40% predicted
	Creatinine >177 umol/L	Creatinine clearance <40 mL/min	Creatinine clearance <40 mL/min
	Cyclophosphamide>6 intravenous courses	Cyclophosphamide cumulative intravenous dose >5 g or >3 months oral	Cyclophosphamide cumulative intravenous dose >3 g/m^2^ or >4 months oral or >6 months intravenous
	–	–	Active GAVE
Mobilisation	Cyclophosphamide 2 g/m^2^, G-CSF	Cyclophosphamide 4 g/m^2^, G-CSF	G-CSF only
Conditioning	Cyclophosphamide (200 mg/kg), rabbit ATG	Cyclophosphamide (200 mg/kg), rabbit ATG	Cyclophosphamide (120 mg/kg), equine ATG
Total body irradiation	No	No	Yes (800 cGy, lung and kidney shielding)
Stem cell manipulation	None	CD34+ selection	CD34+ selection
Comparator arm	Cyclophosphamide 6 monthly intravenous courses (1000 mg /m^2^)	Cyclophosphamide 12 monthly intravenous courses (750 mg/m^2^).	Cyclophosphamide 12 monthly intravenous courses (750 mg/m^2^).
Primary outcome measure	>25% decrease in mRSS, or >10% increase in FVC at 12 months	Survival without new onset heart, lung or kidney failure	Global Rank Composite Score at month 54
Follow-up	2.6 years (mean)	5.8 years (median)	Up to 4.5 years
12-month treatment-related mortality in comparator arm	0 (0%)	0 (0%)	0 (0%)
12-month transplant-related mortality	0 (0%)	8 (10.1%)	1 (3%)

ASSIST, American Scleroderma Stem cell versus Immune Suppression Trial; ASTIS, Autologous Stem cell Transplantation International Scleroderma Trial; ATG, antithymocyte globulin; FVC, forced vital capacity; GAVE, gastric antral vascular ectasia; G-CSF, granulocyte-colony stimulating factor; LVEF, left ventricular ejection fraction; mRSS, modified Rodnan skin score; PAP, pulmonary arterial pressure; SCOT, The Scleroderma Cyclophosphamide Or Transplantation; SSc, systemic sclerosis.

The first RCT was the American Scleroderma Stem cell versus Immune Suppression Trial (ASSIST), a phase II trial comparing autologous non-myeloablative HSCT with monthly intravenous cyclophosphamide therapy.^13^ After randomisation, 10 patients underwent autologous HSCT, while 9 patients in the control group received 6 monthly pulses of cyclophosphamide. At month 12, all patients treated with HSCT demonstrated significant improvement (defined as a>25% decrease in mRSS for those with initial mRSS >14 or a>10% increase in FVC), while 8 of the 9 controls exhibited disease progression (defined as >25% increase in mRSS or >10% decrease in FVC). Of the eight controls with an unsatisfactory response, seven crossed over to the transplant arm, all of whom demonstrated improvement. At 6 months, the annualised rate of change from pretreatment FVC was 34% in patients undergoing HSCT, compared with –10% in controls (p=0.002), at 12 months the rates were 15% and –9%, respectively (p=0.006). Twelve months following randomisation, the mean mRSS had decreased by almost half in the transplant group, but increased in the control group. Similarly, significant and clinically meaningful improvement of HRQoL occurred in the HSCT group with a change in the total 36-item Short Form Health Survey (SF-36) score from 39 to 56 during the 12 months after transplantation, but deteriorated significantly from 50 to 40 in the control arm. After a mean follow-up of 2.6 years, 15 of the 17 patients undergoing HSCT maintained persistent improvement in mRSS and FVC. There were no deaths in either arm of the ASSIST trial. The study had limitations: due to the small sample size, baseline data were not matched, for example, higher baseline mRSS was reported in the treatment group favouring spontaneous regression to the mean. It was also a one centre trial not necessarily reflecting real-world circumstances.

These limitations were addressed in the second trial, the Autologous Stem cell Transplantation International Scleroderma Trial (ASTIS) trial.^14^ This first phase III trial in SSc randomised a total of 156 patients (mean age 44 years) to receive autologous HSCT or 12-monthly pulses of intravenous cyclophosphamide. In the transplant arm of ASTIS, stem cells were mobilised with a total of 4 g/m^2^ of intravenous cyclophosphamide and G-CSF. After conditioning with 200 mg/kg of intravenous cyclophosphamide, administered with hyperhydration and rabbit ATG, CD34-selected stem cells were reinfused (table 1). The majority of the patients in ASTIS (87%) had lung involvement; the mean mRSS at entry was 25, and 10% had severe skin involvement without internal organ involvement. The primary endpoint was event-free survival (EFS), defined as the time in days from randomisation until the occurrence of death or major organ failure. In the group undergoing HSCT, there were better event-free and overall survival rates. During a median follow-up of 5.8 years, 19 deaths and 3 irreversible organ failures occurred in the HSCT group, while in the control group 23 deaths and 8 irreversible organ failures were recorded. In the control arm, an additional seven patients died subsequent to irreversible organ failure. Secondary endpoints of ASTIS, defined as the change in mRSS in the first 2 years, Health Assessment Questionnaire (HAQ), EuroQoL or SF-36 scores were also significantly better in the HSCT group. No significant changes were seen for left ventricular ejection fraction or diffusing capacity of the lung for carbon monoxide (DLCO). However, a 6.3% increase in FVC and a modest but statistically significant decrease in creatinine clearance was seen in the HSCT group. In ASTIS, HSCT was associated with a relatively high treatment-related mortality in the first year after transplantation, higher than that in the other RCTs. In the first year following randomisation, there were 11 deaths (13.9%) in the HSCT group vs 7 (9.1%) in the cyclophosphamide group. Eight of the 11 deaths (10%) in the HSCT group were treatment-related, and no death was attributed to treatment in the cyclophosphamide control arm. In subsequent years, the high treatment-related mortality observed in the first year post-HSCT was outweighed by a significant long-term all-cause mortality benefit observed in the HSCT arm. Between 12 and 24 months following HSCT, SSc relapsed in 22.4% of patients, but significantly fewer patients in the HSCT group as compared with the control group required immunosuppressive medication (22% vs 44%).

The Scleroderma Cyclophosphamide Or Transplantation (SCOT) trial is the third and most recent RCT examining the effects of HSCT in SSc.^15^ Like the ASSIST and ASTIS trials, SCOT compared autologous HSCT with monthly cyclophosphamide pulses; the conditioning regimen, however, differed in that it added TBI. The rationale for irradiation is based in part on the observed higher effectiveness of the transplantation procedure with addition of irradiation in animal models.^33^ The SCOT trial also differed in that it did not employ cyclophosphamide for mobilisation and used less cyclophosphamide during conditioning compared with the previous trials (table 1). Seventy-five patients with SSc (mean age 46 years) were randomised, with 97% having pulmonary involvement, characterised by a mean baseline FVC and DLCO of 74% and 53% predicted, respectively and a baseline mRSS of 30. Fifty-nine patients had received disease modifying antirheumatic medication prior to randomisation. The primary endpoint of the SCOT trial was a Global Rank Composite Score (GRCS), a tool that simultaneously accounts for multiple disease manifestations based on the following hierarchy of outcomes: death, EFS, FVC, HAQ-Disability Index (HAQ-DI) and mRSS. The GRCS does not measure disease activity or severity but compares patients by means of hierarchical ordered outcomes. The GRCS score at 54 months showed superiority of HSCT over cyclophosphamide. Superiority of HSCT was also demonstrated for all-cause mortality; of the 36 patients randomised to receive HSCT, 3 patients had died by month 54 and 1 death was considered treatment-related, while of the 39 patients in the cyclophosphamide arm, 11 died and no death was considered treatment-related. HSCT was also superior to cyclophosphamide in terms of mRSS evolution, change in FVC and HRQoL measures (HAQ-DI and SF-36). No pulmonary arterial hypertension or congestive heart failure was observed in the HSCT arm 54 months after HSCT, significantly less than the 15% and 12% of cases, respectively, observed in the cyclophosphamide arm. In the HSCT arm, 9% of participants required disease modifying antirheumatic drugs (DMARDs) post-transplantation, significantly less than the 44% of patients in the cyclophosphamide arm. There were similar rates of infections (of any grade) in both groups; however, the rate of grade 3 infections or higher per person-year was nominally higher in the transplantation group than in the cyclophosphamide group (0.21 vs 0.13 p=0.09). One patient in the HSCT arm and five patients in the cyclophosphamide arm experienced renal crisis during the 54 months follow-up period. The 54 months post-treatment EFS was 72.2% in HSCT group and 48.7% in control group. The overall survival of autologous HSCT-treated patients was 91% and 77% in the control patients.

In summary, all three RCTs reported significant improvement in organ specific manifestations, such as skin and pulmonary function, and HRQoL. Despite an elevated risk of treatment-related mortality early after the intervention, there were significant long-term survival advantages following HSCT in both of the two RCTs investigating long term survival.

### Risks of HSCT

The benefits of any treatment procedure need to be weighed against its risks. The mortality associated with HSCT appears to be significant in the RCTs, especially in the ASTIS trial. There was no centre effect of mortality in ASTIS.^14^ The small sample size of the ASSIST trial and the selection of patients may explain the survival of all patients in this trial; similarly, the smaller sample size in SCOT than in ASTIS may contribute to differences in mortality. Further, in ASTIS, the solitary variable shown in a posthoc analysis to impact differences in EFS and overall survival was smoking status, as in former/current smokers HSCT lacked the benefit demonstrated in the never-smoking subjects. Similarly, SCOT data also suggest that in former and current smokers, transplantation had no advantage over cyclophosphamide.^15^

The EBMT analysed mortality after autologous HSCT for severe autoimmune disease from 1996 to December 2007 and reported a 5% mortality by day 100 following transplantation.^22^ In 175 patients with SSc transplanted from 1996 until 2007, the mortality was slightly higher (6%) than in other autoimmune diseases, possibly due to the severity of SSc and the presence of major organ dysfunction in transplanted patients with SSc.^22 26^ The causes of death included SSc recurrence (23 patients), transplant-related mortality (12 patients), cardiotoxicity (1 patient), haemorrhage and secondary malignancies (2 patients each) as well as infections (4 patients).^22^ Careful patient selection is crucial in order to reduce treatment-related mortality. SSc has unique and complex cardiac manifestations and cyclophosphamide is associated with cardiotoxicity.^26^ A comprehensive pretransplant cardiac assessment is therefore recommended even in patients without cardiac symptoms and typically includes transthoracic echocardiography, cardiac MRI with gadolinium contrast, a Holter ECG and right heart catheterisation (RHC).^34^ A 6 min walk test is also recommended.^26^ Some authors and the EBMT suggest an intravascular fluid challenge during RHC (10 mL saline/ kg body weight, given intravenous over 10 min).^26 34^ A mean pulmonary arterial pressure (PAP)>30 mm Hg or a systolic PAP>40 mm Hg after fluid challenge could detect patients with subclinical heart involvement related to cardiomyopathic restriction seen in SSc, who might fluid-sensitive and develop complications during hypervolemic interventions often necessary during the conditioning regimen or should systemic infection develop.^26 34^

HSCT can induce gonadal failure in both sexes. In men, azoospermia occurs in the majority of patients but in most cases testosterone levels remain within the normal limits.^35^ In premenopausal women, the conditioning regimen results in transitory or permanent amenorrhoea with concomitant infertility and menopausal symptoms. Before mobilisation and HSCT, consideration should therefore be given to infertility in both sexes (semen, oocyte or embryo cryopreservation as appropriate). Hormone replacement therapy should be started with gonadal failure.^36^ TBI used as part of the conditioning regimen plays a central role in post-transplantation infertility,^35^ but in regimen without TBI a substantial percentage of women have been described to remain fertile, give birth to healthy babies and without increased occurrence of miscarriages.^37^ The decline of post-HSCT rates of sexual activity and diminished interest/libido and adequate function is possibly related to preparation regimen using alkylating agents and TBI which impacts on the function of the hypothalamic-pituitary-gonadal axis function. Sexual dysfunction may persist long term in both sexes despite some recovery after the 6 months post-transplant nadir.^38^

Regarding renal protection and outcome, life-long post-transplant therapy with angiotensin-converting enzyme inhibitors or angiotensin receptor blockers is recommended to prevent renal crisis.^26 39^ However, it is unclear whether prophylactic ACE inhibition leads to more severe outcomes of renal crisis.^40^

In addition to better understanding frequency of and predisposition to relapse/graft failure, long-term follow-up of transplanted patients with SSc will be important to better characterise and quantify known late sequelae of HSCT, such as secondary autoimmune diseases and secondary malignancies.^41^ Five years after autologous HSCT for autoimmune diseases, the cumulative incidence of secondary autoimmune diseases was as high as 9.8%.^42^ Intriguingly, the ‘new’ autoimmunity developing after transplantation appeared to be mainly antibody-associated and organ-speciﬁc.^43 44^ Lupus erythematosus as primary autoimmune disease and ATG use were risk factors for the occurrence of secondary autoimmune diseases, whereas the presumed beneficial role of CD34+ graft selection is controversial.^42 44^ The attenuation of immunological memory in the B cell compartment after autologous HSCT also implies that patients must be reimmunised.^45^

Because of the high risk of treatment related side effects and of early treatment related mortality, the new EULAR treatment recommendations advice for careful selection of patients with SSc for HSCT. It is also highlighted that the experience of the medical team are of key importance.^4^

### Research agenda

Head-to-head studies have not been carried out to determine the optimal stem cell collection and conditioning procedure in the HSCT regimen. It is unclear, if the ex vivo CD34+ selection of stem cells truly confers a benefit over non-selected cells.^16^ The potential risk of reinfusing autoreactive T-cells with non-selected grafts may be outweighed by reduced numbers of transplanted cells and increased immunosuppression in patients receiving CD34+ grafts.^46^ Reducing cyclophosphamide doses may on the one hand decrease toxicity in terms of infections, but on the other hand decrease the efficacy of HSCT. Cyclophosphamide may also induce haemorrhagic cystitis. Patients should receive urometixan.^36^ It is however unclear if the hyperhydration recommended for the prophylaxis of haemorrhagic cystitis^36^ may contribute to transplant related mortality in patients with decreased cardiac compliance.

Animal studies indicate that a conditioning regimen that adds irradiation to cyclophosphamide may provide a better control of autoimmune diseases.^33^ As the SCOT trial has inclusion criteria and a control group similar to those in the ASTIS trial, a comparative analysis with regard to the use and not-use of irradiation during conditioning may be possible. In the SCOT trial, only 9% of transplant recipients had initiated DMARDs by 24 months, as compared with 22% in the ASTIS trial, suggesting better SSc control with myeloablative HSCT. Adding TBI, however, also implies an excess risk of cancers over a lifetime.

With regard to the known cardiotoxicity of high-dose cyclophosphamide used during the conditioning step, it is unclear which patients are at particular risk and which conditioning is best. In order to diminish cyclophosphamide requirements, conditioning regimens have been used that employ thiotepa, a non-cardiotoxic alkylating agent,^47^ but comparative studies are lacking.

It is also currently vague, if other agents used during the HSCT procedure put specific patients with SSc at risk. G-CSF could induce disease flares if administered alone, although the combination with cyclophosphamide in the mobilisation step effect may prevent flares and improve stem cell yields.^36^ ATG is associated with a risk of allergic reactions and its profound immunosuppression increases the risk of acquired and reactivated infections.^36^

To address the issue of disease relapse after successful transplantation, the Scleroderma Treatment with Autologous Transplant (STAT) trial (ClinicalTrials.gov identifier: NCT01413100) is currently recruiting. The conditioning regimen of STAT uses no CD34+ selection after mobilisation of stem cells and apheresis and the conditioning regimen consists of cyclophosphamide and ATG, but without TBI. In the STAT trial, open label mycophenolate mofetil is added approximately 2–3 months post-transplant after the stem cells have ‘engrafted’ and mycophenolate mofetil is maintained for 2 years. The primary outcome of this single group trial is defined as EFS 5 years after transplantation.

Last, developing more specific and validated criteria for patient selection is ongoing. It is currently unclear which cardiac screening is optimal and which cardiac parameters are predictive of an increased transplant related cardiac mortality. Hopefully, analysis of past and future studies will help clarify risk stratifications incorporating factors of age, functional performance status, rate of disease progression, parameters of SSc damage, smoking and cardiac compliance.

## Summary and conclusion

Autologous HSCT is the first therapeutic intervention with proven survival advantages in a subgroup of patients with SSc. In addition to improving survival, three RCTs demonstrated significant improvement of organ function, vasculopathy, skin involvement and HRQoL. HSCT has induced clinically meaningful and sustained improvements of lung function despite decline in control groups. Autologous HSCT is currently the only disease modifying strategy with high-level evidence for improving SSc survival, prevention of organ worsening and improvement of pulmonary function. Although autologous HSCT offers the possibility of drug-free remission, patients may experience a relapse requiring disease modifying antirheumatic drugs. HSCT is also associated with secondary autoimmune diseases and gonadal failure. The benefits of HSCT must be balanced against the increased risk of transplant-related mortality in the first year. A patient with early SSc, internal organ involvement and concomitant poor prognostic factors has a limited window within which they are eligible for HSCT. As the disease progresses, severe internal organ damage ensues and constitutes an exclusion criterion for the procedure. The EULAR treatment recommendations indicate HSCT for carefully selected patients with rapidly progressive SSc at risk of organ failure. HSCT should be performed in centres experienced in HSCT. More specifically, we recommend considering HSCT in patients with early, progressive diffuse SSc with strongly reduced survival as predicted by validated tools, yet without severe organ involvement.^48^
